# Cardiac Fibrosis Automated Diagnosis Based on FibrosisNet Network Using CMR Ischemic Cardiomyopathy

**DOI:** 10.3390/diagnostics14030255

**Published:** 2024-01-24

**Authors:** Mohamed Bekheet, Mohammed Sallah, Norah S. Alghamdi, Roxana Rusu-Both, Ahmed Elgarayhi, Mohammed Elmogy

**Affiliations:** 1 Applied Mathematical Physics Research Group, Physics Department, Faculty of Science, Mansoura University, Mansoura 35516, Egypt; 2 Radiography and Medical Imaging Department, Faculty of Applied Health Sciences Technology, Sphinx University, New Assiut 71515, Egypt; 3 Department of Physics, College of Sciences, University of Bisha, P.O. Box 344, Bisha 61922, Saudi Arabia; 4 Department of Computer Sciences, College of Computer and Information Sciences, Princess Nourah bint Abdulrahman University, P.O. Box 84428, Riyadh 11671, Saudi Arabia; 5 Automation Department, Faculty of Automation and Computer Science, Technical University of Cluj-Napoca, 400027 Cluj-Napoca, Romania; 6 Information Technology Department, Faculty of Computers and Information, Mansoura University, Mansoura 35516, Egypt

**Keywords:** fibrosis, CMR, magnetic resonance imaging, LGE, deep learning, FibrosisNet

## Abstract

Ischemic heart condition is one of the most prevalent causes of death that can be treated more effectively and lead to fewer fatalities if identified early. Heart muscle fibrosis affects the diastolic and systolic function of the heart and is linked to unfavorable cardiovascular outcomes. Cardiac magnetic resonance (CMR) scarring, a risk factor for ischemic heart disease, may be accurately identified by magnetic resonance imaging (MRI) to recognize fibrosis. In the past few decades, numerous methods based on MRI have been employed to identify and categorize cardiac fibrosis. Because they increase the therapeutic advantages and the likelihood that patients will survive, developing these approaches is essential and has significant medical benefits. A brand-new method that uses MRI has been suggested to help with diagnosing. Advances in deep learning (DL) networks contribute to the early and accurate diagnosis of heart muscle fibrosis. This study introduces a new deep network known as FibrosisNet, which detects and classifies fibrosis if it is present. It includes some of 17 various series layers to achieve the fibrosis detection target. The introduced classification system is trained and evaluated for the best performance results. In addition, deep transfer-learning models are applied to the different famous convolution neural networks to find fibrosis detection architectures. The FibrosisNet architecture achieves an accuracy of 96.05%, a sensitivity of 97.56%, and an F1-Score of 96.54%. The experimental results show that FibrosisNet has numerous benefits and produces higher results than current state-of-the-art methods and other advanced CNN approaches.

## 1. Introduction

Heart disease comes in various forms, and each has unique signs, causes, and treatments. Some people’s health can significantly improve by lifestyle modifications and taking medications. Others could require surgery to restore the functionality of their tickers. There are several types of heart disease, including cardiovascular disease (CVD), heart arrhythmias, heart failure, heart valve disease, pericardial disease, and cardiomyopathy (heart muscle disease). CVD is approximately half of any communicable diseases (NCDs). Although CVD continues to be the most significant cause of death globally, accounting for 17.3 million deaths per year, that number is anticipated to increase to >23.6 million by 2030. NCDs already outnumber communicable diseases as the most significant cause of global disease burden [1,2].

Until now, professionals have devised several screening methods for diagnosing CVDs. The most important techniques for the diagnosis of CVDs include ECG [3], Echo [4], exercise stress test [5,6], carotid ultrasound [7], CT scan [8], and cardiac magnetic resonance (CMR) imaging [9,10]. Based on its merits, CMR imaging has recently been acknowledged as one of the specialized doctors’ best diagnostic methods for CVDs [9,10].

Cardiac fibrosis is heart muscle disease; cardiovascular disease is the leading cause of death worldwide [11]. One of the most frequent histologic characteristics linked to myocardial damage is myocardial fibrosis (MF) [12]. CVD is the largest cause of death globally. CVD and ischemic heart diseases are closely related [13]. These patients have permanent cardiac necrosis due to ischemia, replacing the contractile muscle with a kinetic scar [14].

This naturally lowers cardiac function and results in heart failure. Myocardial fibrosis comes in two major forms. The initial kind of arterial hypertension, valvular heart disease, diabetic cardiomyopathy, hypertrophic cardiomyopathy, idiopathic-dilated cardiomyopathy, and heart aging all exhibit reactive interstitial fibrosis. It occasionally has a localized perivascular distribution and a diffuse microscopic distribution in the myocardium.

The second type is replacement fibrosis, which usually develops following myocyte damage or demise, primarily in severe ischemia circumstances, when cells undergo apoptosis, stimulate fibroblasts, and encourage the myocardium to have collagen-rich fibrous tissue deposited there [15]. Among these, one-third of individuals also pass away abruptly, resulting from harmful ventricular arrhythmias (VA). They are often caused by electrical reentry circuits close to cardiac fibrosis regions. The accepted reference standard for myocardial scar imaging is magnetic resonance imaging (MRI) in two dimensions (2D) with late gadolinium enhancement, distinguishing the latter with the retention of gadolinium-based contrast compounds as an enhancement [16].

However, because of hardware and software improvements, the electrophysiology community has expressed interest in using these pictures to guide therapies, and the application of three-dimensional (3D) capture is quickly expanding [17]. CMR is particularly good at identifying localized apical hypertrophy, usually missed by echocardiography and cardiac hypertrophy in HCM patients. CMR can be utilized to find myocardial fibrosis as well. Along with myocardial fiber disarray, myocardial fibrosis in HCM patients may serve as a significant substrate for the emergence of ventricular arrhythmias. Using contrast media based on gadolinium agents enables the high spatial resolution detection of cardiac fibrosis by CMR [18].

A noninvasive method to evaluate cardiac fibrosis needs to be able to describe soft tissue and have great temporal and spatial resolution. Therefore, CMR is the preferred imaging technique. Although CT has also been investigated [19], it is less effective than CMR for characterizing soft tissues. We will concentrate on late gadolinium enhancement (LGE) as a technique for enhancing gadolinium, the most popular CMR method for fibrosis imaging.

CMR is a technology that creates finely detailed images of the heart and its environs using radio waves, a powerful magnetic field, and a computer. For individuals with congenital heart defects or other heart disorders that occur after birth, cardiac magnetic resonance imaging (CMR) is used to detect and track heart disease as well as to assess the various cardiac components and their functions. Because CMR uses no ionizing radiation to obtain images, unlike CT, it is a safe alternative that is especially beneficial for expectant mothers. However, it is advised to postpone having it performed until after the fourth month of pregnancy.

Cardiac tissue can be described by CMR. More specifically, the late gadolinium enhancement method can be used to identify localized fibrosis patches. Different patterns in the distribution of this fibrosis allow one to distinguish between ischemia cardiomyopathy (iCMP) and non-ischaemic cardiomyopathy (nCMP), and in some situations, to identify the underlying cause of the latter. The parametric T1 mapping sequences can also be utilized to detect diffuse fibrosis. Because of this, the extracellular volume is either calculated following the administration of the contrast agent (c.a.) or the tissue’s native T1 is evaluated before c.a. Both diffuse and localized fibrosis appear to be useful prognostic markers for identifying potentially harmful ventricular arrhythmias and sudden cardiac death when evaluated with CMR.

Cardiovascular imaging modalities (echocardiography, single-photon emission computer tomography (SPECT), positron emission tomography (PET), multi-detector computer tomography (MDCT), and cardiovascular magnetic resonance (CMR)) play important roles in the diagnosis, management, and prognostic assessment of cardiac diseases because of their ability to detect pathophysiological myocardial changes like fibrosis, edema, and infiltration to varying degrees. CMR, in addition to being better in the noninvasive characterization of cardiac tissue, outperforms different imaging techniques in terms of its multipara metric together with a thorough heart evaluation (central example). CMR is accurate in assessing cardiac architecture and function.

Recently, the term “artificial intelligence” (AI) has gained popularity due to disruptive technology advancements and exceptional experimental outcomes, particularly in image processing and analysis. Medical specialties requiring significant images, like imaging and pathology, have seized the chance and made significant investments to fully utilize the promise of AI. It has increasingly been used for routine medical image analysis tasks like classification, segmentation, and diagnosis [20].

The last ten years have seen much interest in AI healthcare techniques. Processing medical images on computers has undergone a revolution. Massive increases in computing power have accompanied advancements in machine learning (ML) approaches, especially the introduction of imaging and deep learning (DL). Precision medicine, clinical predictive modeling, and image analysis are all areas in which ML and DL are quite adept [21].

Computerized illness detection, histology, description of phase or subgroup, and sorting of patients according to treatment results or prediction are all made possible by applying AI to medical images. Additionally, it enables highlighting particular areas in images, extracting attributes from images, and calculating component volumes. It results in measuring characteristics or picture quality when machine learning algorithms are used in classification [22].

In this study, we develop an automated DL method for CMR fibrosis diagnosis using histopathology of 3420 images. The key contributions of our suggested system are outlined in the following points:

1. The training process at FibrosisNet has been given sufficient time.
2. Three convolutional layers with tiny kernels are utilized to reduce the training parameters.
3. Several performance metrics are used to assess the proposed system. Additionally, we validated our proposed system by contrasting it with a few already-in-use solutions.
4. Compared to transfer learning methods, FibrosisNet attained the highest level of accuracy.

The remaining five sections of the paper will be arranged as follows. Section 2 talks about the ongoing related works of cardiac fibrosis detection. The proposed approach is presented in Section 3. The results of the study and the analysis are detailed in Section 4. Lastly, the conclusion is presented in Section 5.

## 2. Related Work

Researchers have always been impacted by the nature of medical imaging data when creating deep learning-based diagnosis and prognosis systems [23]. The most prevalent types of medical imaging data include histopathology slides, X-rays, CT scans, endoscopic pictures, and MRIs [24,25]. In several sectors, DL is currently surpassing other traditional machine learning approaches. DL methods have consistently performed exceptionally well in various fields, including medical imaging. Using image processing techniques made it simpler to identify and categorize diseases.

### 2.1. Machine Learning Techniques

Campese et al. [26] assessed a cohort of subsequent CMR experiments using two alternative Machine Learning (ML) algorithms, namely kernel methods with Support Vector Machine (SVM) and Convolutional Neural Network (CNN). The objective was a binary classification task that determined whether a cardiac scar was present. The best results obtained an accuracy of 71% and a sensitivity of 72%.

Dima et al. [27] discussed creating a classification system to analyze ECG and VCG records to diagnose heart scarring. Four distinct methodologies have been investigated to develop a set of markers that will allow for the differentiation between scar presence and absence. Their overall accuracy was 82.07%, while their sensitivity and specificity levels were 76% and 87.5%.

Zabihollahy and Ukwatta [28] used CNN and created a model using machine learning and deep learning to assess heart fibrosis depending on thirty patients’ datasets. Automated dividing and quantification of MF is crucial. This study intends to discuss ML-based techniques created for MF measurement in the LV using CMR images, and the DSC was 88.61%.

### 2.2. Deep Learning Techniques

Asif et al. [29] introduced fully automatic analysis produced from AI on normal, unfiltered, and ungated CT lung examinations with contrast enhancement. LV dilatation is possible. Compared to CMR, they developed standards for identifying signs of LV enlargement on CT, which might be utilized to systematically check for stretch heart conditions at the moment of the CT. This technique’s maximum documented accuracy was 71.9%.

Shark et al. [30] designed the four heart chambers and great vessels have been fully automated, accurately, and with a low failure rate in CTPA. DL volumetric indicators may enhance invasive hemodynamic prediction and CTPA cardiac assessment. They employed a rigorous testing technique to assess the model and show that it can be applied to hospitals and CT suppliers with various acquisition processes. This technique’s best accuracy was 93%.

Penso et al. [31] constructed a deep-learning model to identify heart fibrosis depending on fifty patients’ datasets. Without further contrast agent administration or radiation treatment, DL on early CE-CCT capture may enable the assessment of LV sectors impacted by heart fibrosis. A tool like that might decrease user engagement and visual inspection, which would save time and effort. Their results were an accuracy of 71% and a sensitivity of 73%.

Shi et al. [32] constructed a model for correctly determining native LGE T1 mapping. The SE-ResNext-50-based DL framework could be used. Without a contrast agent, it is a potential method for the early diagnosis of cardiac fibrosis in HCM. Using the ResNext-50 algorithm, the model attained an area under the curve AUC of 83%, a sensitivity of 79%, and a specificity of 87%. 

Jafari et al. [33] developed DL-based CADS to identify CVDs from CMR images. The CADS steps with datasets, preprocessing approaches, and DL methods were described. The test outcomes revealed 90.18% accuracy in detecting LV scars. They used the CTAEM-NET algorithm. They also used many implementation tools and evaluation parameters in their study and used 34 subjects’ datasets.

Popescu et al. [34] created and tested a deep neural network for automatic, anatomically correct expert-level LGE-CMR myocardium and heart fibrosis dividing, allowing for direct clinical measure calculation. Given the complexity of the training set, our technique might be applied to various imaging modalities and patient illnesses. They asserted to have accuracy rates of 96% and 75%.

Moccia et al. [35] achieved two separation algorithms that could determine scar tissue in the CMR-LGE images. However, better results were obtained when the search area was restricted to the heart region. The results of this study provide an encouraging starting point for using FCNNs to segregate nonviable scar tissue from CMR-LGE images. They claimed a sensitivity of 88.07% and a Dice similarity coefficient of 71.25%. 

Gumpfer et al. [36] created DL models that have yielded encouraging performance metrics for detecting myocardial scars. They performed this method instantly on ECG data and clinical parameters without acquiring features in advance, allowing for easy and flexible use. They claimed their study had attained an area under the curve score, sensitivity, specificity, and accuracy of 89%, 70%, 84.3%, and 78.0%, respectively, for detecting myocardium scars.

Muthulakshmi and Kavitha [37] suggested that a deep convolutional neural network (CNN) with Levenberg–Marquardt (LM) learning is used to estimate left ventricular (LV) volume from CMR images and perform a mass screening of myocardial ischemia individuals. They achieved an accuracy of 86.39%, sensitivity of 90%, and AUC of 0.93.

Ahmed et al. [38] demonstrated the first proof of concept for quantifying left ventricle (LV) mass and scar volume on left ventricular endoscopy (LGE) in patients with heart failure (HCM) automatically using deep convolutional neural networks (DCN). They achieved segmentation accuracy for the scar, which was 0.57 ± 0.23 (per patient) and 0.58 ± 0.28 (per slice), while for the LV, it was 0.82 ± 0.08 (per patient) and 0.81 ± 0.11 (per slice).

There has been much prior scientific research on identifying fibrosis using different models, but it has significant limitations. Maybe there were several drawbacks, including a smaller dataset with fewer images, less precision, the necessity for manually created elements, a shortage of variety of data with not publicly accessible data, and an unbalanced quantity of images in data. To address the limits mentioned above, we suggest a new fibrosis network. The network model for fibrosis CMR detection used in this study was tailored, unlike some past studies that used a capsule network. The customized model presents all the image processing traits discovered during the training step. As shown in the findings section of this research, we used a customized FibrosisNet and achieved good results. Table 1 summarizes the above-mentioned related studies for heart fibrosis, and their accuracy for automated diagnosis.

## 3. Methodology

### 3.1. Dataset

**Figure 1:**
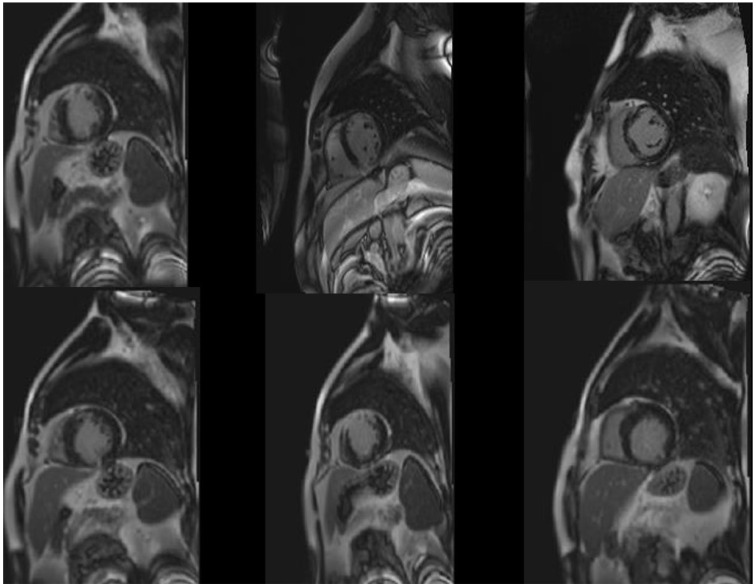
Samples of fibrosis heart images, short axis LGE sequence.

In this retrospective investigation, MRI scans from 500 healthy individuals and 640 patients with clinically diagnosed fibrosis were evaluated under the protocol approved by the local ethics committee of the Faculty of Science, Mansoura University (Code number: Sci-phy-M-2022-106). This retrospective analysis gathered these datasets anonymously as the scans were stored in the DICOM format. Figure 1 displays a sample of fibrosis heart scans, short axis LGE sequence. The dataset has been augmented to ensure the algorithm can recognize samples as new images, which is often used to reduce overfitting and boost model resilience. After the augmentation procedure, the number of images increased to 3420, including 1920 fibrosis images and 1500 normal images. Table 2 divides the dataset into training sets (80%) and test sets (20%).

**Figure 2:**
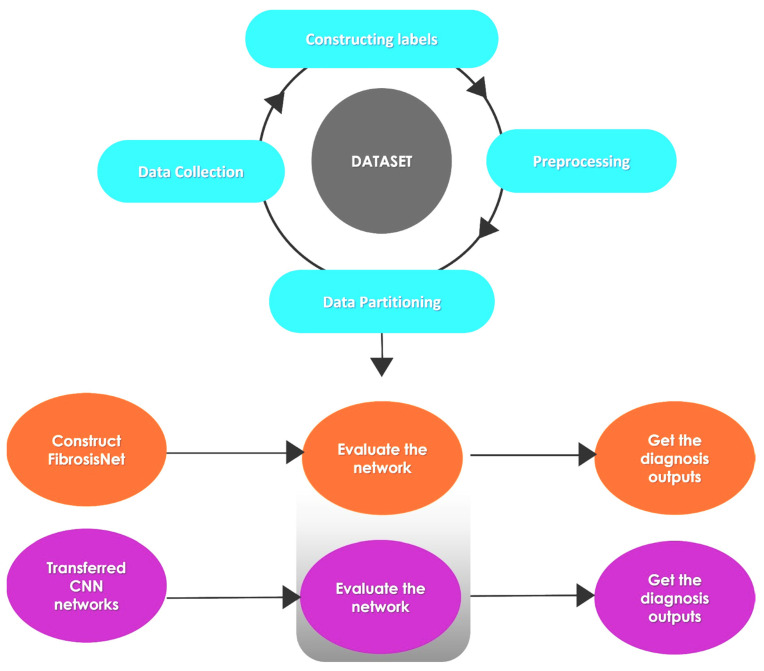
The proposed framework for detecting heart fibrosis.

This section covers the specifics of the proposed CNN model implementations and the architecture of the suggested CNN techniques used to identify fibrosis CMR situations. This study introduces FibrosisNet, a new deep-learning model that uses a convolutional neural network to determine fibrosis in MRI images. The following are three unique improvements that have been made to three separate well-known CNNs, including MobilenetV2, GoogleNet, and ResNet50. The hyperparameters and optimization algorithm are configured after applying the CNN-proposed structural and modification models. The best network structure is then determined using training and performance computations. Figure 2 displays the block diagram of the proposed technique.

### 3.2. Preprocessing

**Figure 3:**
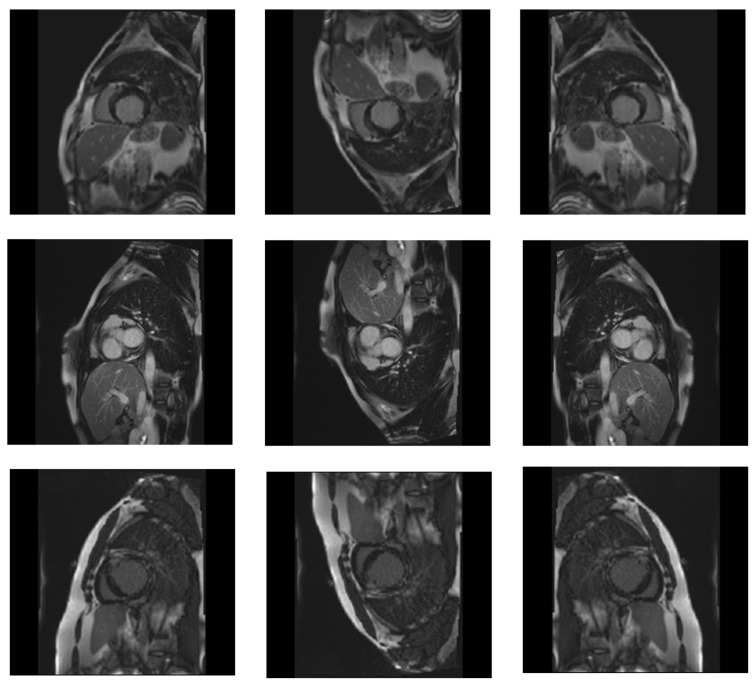
This augmentation was performed using MATLAB’s function. The (**left**) column indicates the original images, the (**middle**) column indicates the vertically augmented images, and the (**right**) column indicates the horizontally augmented images.

The images are preprocessed before being input into the structures. The first step is to minimize the original image from 256 × 256 × 3 pixels to 224 × 224 × 3 pixels to minimize dimensionality, simplify computations, and improve network performance. The grey images (two-channel) are then converted into three-channel images that work with each pre-trained deep network’s input layer. The images from the study are augmented in the second phase so that the systems can identify them as brand-new instances. An augmentation step adding more training data is often used to reduce overfitting on the training dataset and improve prediction accuracy on the testing dataset. As seen in Figure 3, all the initial images are flipped horizontally and vertically. Lastly, the data are split into the initial and evaluation sets, with each category having its own set of training target labels. Eighty percent of the data comes from the initial training set, while twenty percent comes from the experimental set. All the data are used to evaluate the efficiency of the system.

### 3.3. Processing

**Figure 4:**
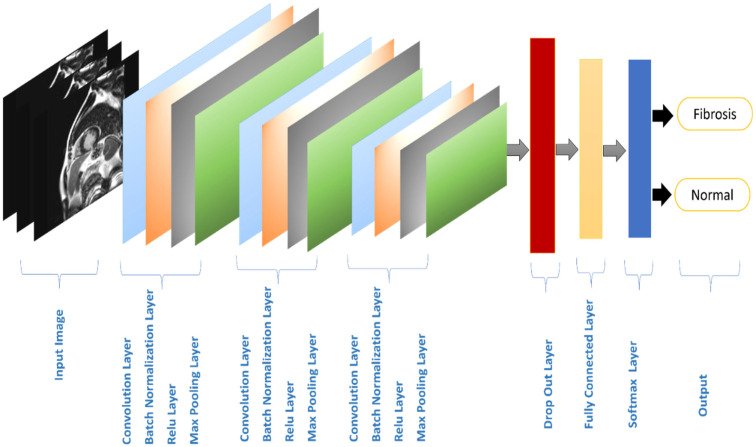
The proposed model architecture.

The proposed model architecture has been displayed in Figure 4. The input layer emphasizes input image size and data normalization execution. This layer has dimensions 224 × 224 × 3. Three convolutional layers are added after that. A sliding kernel or filter (F) matrix with a size of (QR) with the input can be used to discretely convolutionally compute each feature, which together make up a 2D grid. The padding (P) is the number of zeros added to the image’s border, whereas the stride (S) is the slide over the kernel.

The kernels of the first layers determine low-level characteristics like lines and edges. The following, however, are used to identify more intricate traits. The parameters used are F = 5, 5, and 3, Q × R = 5 × 5, 5 × 5 and 3 × 3, S = 1, and the same padding for the convolutional layers 1, 1, 1, and 1, respectively. ReLU, which takes less time to train than other activation functions, is applied to each convolution layer.

Then, three maximum pooling layers are used. Each one splits the entire image into little rectangles of size (4 × 4) with a deliberate step (1 × 1) at the end of every three convolutional layers to downscale. Decide then on the 25 pixels with the highest number. The pooling layer effectively reduces the number of parameters and, consequently, calculations in CNN. By removing some nodes from the network, the dropout layer helps to decrease overfitting while accelerating training. Our suggested model uses a single layer with a 60% dropout rate.

Finally, four advanced layers have been employed—two fully connected layers (FC), one SoftMax layer, and one classification layer. Each neuron in a layer is connected to another neuron in other layers by the FC layer. Two neurons serve as the FC layer’s output in our structure and will eventually serve as placeholders for the two classes. Following that, a SoftMax classifier layer is applied to the advanced FC layer to forecast the classes. Each predicted class is given a value by the SoftMax layer between [0 1], and the total of all the predicted class values equals 100%. The proposed model is a deep learning approach that detects cardiac fibrosis cases. FibrosisNet is a new CNN where it has new layers connection, each layer has some specific parameters. Table 3 shows the layer’s name and number of proposed CNN (FibrosisNet), output shape, and learnable parameters.

### 3.4. Transfer Learning CNN Architectures

Three previously trained networks are tuned in at this stage: MobileNetV2, GoogleNet, and ResNet50. To achieve the best results, some factors have been changed. Three models are produced following modification in each network, for nine models. Each model starts with a 224 × 224 × 3-pixel input layer and goes on to have updated layers.

#### 3.4.1. MobileNet Model

Sandler et al. [39] presented Google’s MobileNetV2 in 2018, which is another effective DCNN-based classifier method. We selected their classifier for this inquiry since it performs well on benchmarks like ILSVRC. MobileNetV2 is a brand-new idea inspired by MobileNetV1 [40]. However, it has two extra features. Multiple layers with linear restrictions are the first characteristic, and providing quick paths for connecting bottlenecks is the second.

Here are three different models employed in the MobileNetV2 network. The number of modified layers in each model has been adjusted. Each model begins with a 224 × 224 × 3-input layer comprising the preprocessed images with 154 layers. The first model reduces the number of outputs from 1000 to 2 on the final fully connected layer (FC). In the second model, an extra layer has been replaced with a new layer. Accordingly, the same procedure has been applied to the last model to obtain three new layers. Furthermore, details about layers’ modifications are shown in Table 4.

#### 3.4.2. GoogleNet Model

A deeper and wider CNN model has been identified as GoogleNet [41], the 2014 ImageNet competition winner. GoogleNet won that competition with a top-five error rate of 6.67%. The network GoogleNet implements three distinct structures. A distinct number of layers have been altered in each model. Each model begins with an input layer sized 224 × 224 × 3 and consists of 144 layers. In the initial model, the number of outputs in layer 142, which corresponds to the last FC layer, is modified to have two outputs. In the second model, a new layer has been inserted to replace layer 141. Similarly, the approach is used in the final model with layers 142–140, maintaining the utilization of the SoftMax classifier in all the models. The summary of the layer adjustments for all models in a tabular format in Table 5.

#### 3.4.3. ResNet Model

He et al. [42] introduced the ResNet architecture in 2015. They trained deep convolutional networks using the conventional stochastic gradient descent (SGD) approach via residual modules. We use the ResNet50 model in our study because it is one of the most well-liked ResNet models overall and because it has a simpler structure than the other varieties. Three different models are used in the ResNet50 network. Each model has 177 levels and starts with an input layer of 224 × 224 × 3. The first model replaces layer 175’s outputs with two outputs. In the second model, an additional layer with the number 174 has been substituted with a new layer. The final model was processed similarly until three more layers were added. The classification SoftMax layer has not been altered in all three models. The details of the layers’ modifications are shown in Table 6.

## 4. Experimental Results

### 4.1. Software and Hardware Configuration

The equipment used for the work has the following features: Dell Workstation 7910 with two Intel Xenon E2686v4 18C 36 MB caches. It has 256 GB DDR4 RAM and an NVIDIA RTX 3060 with 12 GB VRAM on 64-bit Windows. 

### 4.2. Evaluation Metrics

Some assessment measures are used to evaluate the effectiveness of the classifier. Various calculations have been introduced to calculate the evaluation metrics. The true positive (*TP*) value is a numerical measure that represents the accurate identification of a fibrosis case. The value of (*FP*) measures the number of inaccurate results, which denotes the incorrect detection of fibrosis. The quantity (*TN*) represents the numerical value that accurately denotes the correct detection of normal samples as a true negative. The value of (*FN*) measures the number of inaccurate results, which denotes the incorrect detection of instances belonging to the normal class.

The inclusion of the confusion matrix components is essential for the computation of accuracy (ACC), sensitivity (SEN) (also known as recall), and specificity (SPC). In addition, the F-measure coefficient (F1-score), Matthew correlation coefficient (MCC), positive predictive value (PPV), and negative predictive value (NPV) are used. PPV is also known as precision. The performance metrics utilized in this study are presented in the following equations.

(1) $$\mathrm{ACC}\left(\%\right)=\frac{TP+TN}{TP+TN+FP+FN}\times 100$$

(2) $$\mathrm{SEN}\left(\%\right)=\frac{TP}{TN+FP}\times 100$$

(3) $$\mathrm{SPC}\left(\%\right)=\frac{TN}{TN+FP}\times 100$$

(4) $$\mathrm{PPV}\left(\%\right)=\frac{TP}{TP+FP}\times 100$$

(5) $$\mathrm{NPV}\left(\%\right)=\frac{TN}{TP+FP}\times 100$$

(6) $$F1\text{-}\mathrm{score}\left(\%\right)=\frac{2\left(\mathrm{Precession}\times \mathrm{Recall}\right)}{\mathrm{Precession}+\mathrm{Recall}}\times 100$$

(7) $$\mathrm{MCC}\left(\%\right)=\frac{TP\times TN-FP\times FN}{\sqrt{\left(TP+FP\right)\left(TP+FN\right)\left(TN+FP\right)\left(TN+FN\right)}}\times 100$$

### 4.3. Results

This section presents the experimental analyses that demonstrate the obtained findings. Ten models have been trained on the dataset for fibrosis detection. A total of nine different models of pre-trained CNNs, including MobileNetV2, GoogleNet, and ResNet50, are presented. In addition, FibrosisNet is a new proposed CNN, as mentioned in the methodology. FibrosisNet has a higher accuracy. Table 7 presents a comprehensive evaluation of fine-tuning techniques applied to three deep networks and FibrosisNet. For the MobileNetV2, model 1 provides the optimum performance. The accuracy achieved in the experiment is 87.13%. The sensitivity of the result is 85.07% due to a false negative rate of 63.

**Figure 5:**
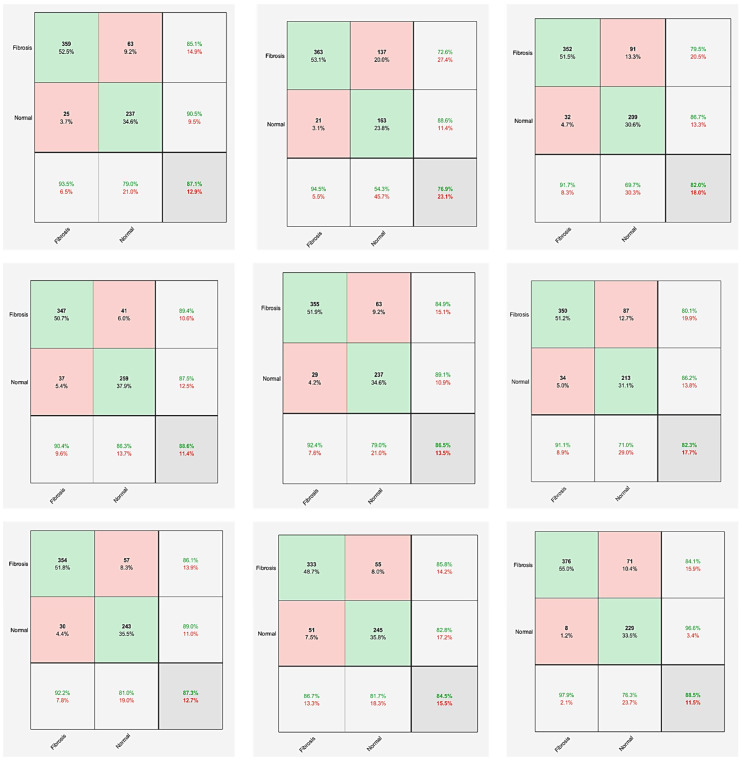
The confusion matrix for each modified model for all networks. The first row indicates the MobileNetV2 models. The second row indicates the GoogleNet models. The third row indicates the ResNet50 models.

Additionally, due to the occurrence of false positives being 25, the resulting specificity value is calculated to be 90.46%. This model can predict the fibrosis class more than the normal, as the PPV value is 93.49, while the normal is equal to 97%. Model 1 of the GoogleNet network demonstrates a high level of accuracy with 88.60%. The SEN, SPC, and F1-Score values are 89.43%, 87.5%, and 89.9%, respectively. The ResNet50 network has the highest accuracy of 88.45% in model 3. Furthermore, it achieves a PPV percentage of 97.92% and an F-Score of 90.49%. Figure 5 shows the confusion matrix for each modified model for all CNNs.

**Figure 6:**
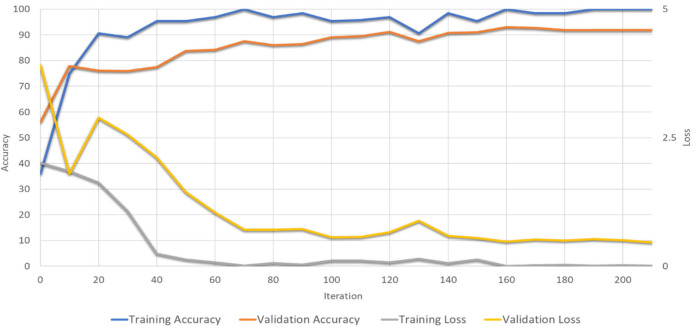
Training and testing for the optimum FibrosisNet architecture.

**Figure 7:**
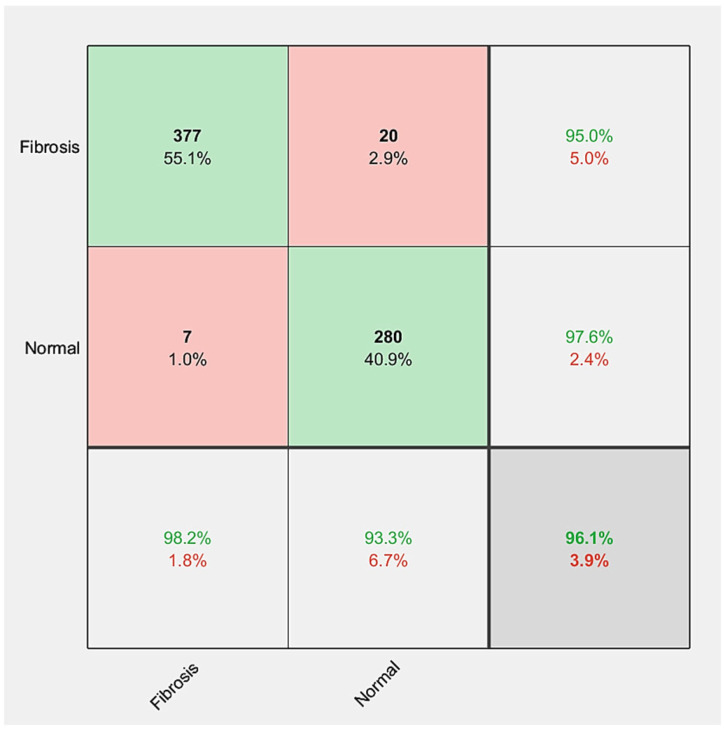
The FibrosisNet confusion matrixes for the tested fibrosis and normal images.

Finally, the FibrosisNet demonstrates superior performance compared to the other networks. The performance results of the FibrosisNet are shown in Figure 6. The training accuracy reaches the maximum value at the end of iterations, while the testing accuracy reaches the value of 96.05%. The obtained sensitivity is 94.96%, while the specificity is 97.56%. Moreover, the training loss reaches the minimum value at the end, while the testing loss reaches the value of 0.2 at the end of iterations. The confusion matrix shown in Figure 7 illustrates the performance evaluation of the FibrosisNet model, which has been decided to be the most optimal.

### 4.4. Discussion

This research proposes a method for detecting fibrosis using magnetic resonance images by introducing a new CNN named FibrosisNet network. In addition, nine different CNN models based on transfer learning are applied. A large set of parameters is employed to fine-tune the networks to produce an effective outcome for each of the suggested models for the CNNs. The extracted results showed that the FibrosisNet network is the best among the other applied networks. It achieves an accuracy of 96.05% and a precision of 98.18%. Incorrect diagnosis and failure to detect the disease early leads to negative repercussions for the patient. The proposed model solved the problems because it has small FN and FP values, which leads to a high value of accuracy and sensitivity. A diagnostic test is considered FP if it reports an illness in a patient who does not truly have it. In a similar vein, a test result is a false negative if it indicates that the patient has an illness that is definitely absent. False positive and false negative test results both suggest that the observed condition differs from the true one.

FP means that the machine predicted that these images were fibrosis, but what is correct is that these images are normal. FN means that the machine predicted that these images are normal, but what is true is that these images express fibrosis. Therefore, FN is more dangerous, and it has greater repercussions for the patient.

In the FibrosisNet, the value of the FN is 20 images. This means that we have 20 images of fibrosis, and the machine predicted that these images are normal. The value of the FP is 7, which means that we have normal images, and the machine incorrectly predicted that these are images of fibrosis, and thus the rate of correct diagnosis is high, which helps in early detection of the disease.

According to the models of the existing CNN networks, the first model, GoogleNet has the highest accuracy because the number of FP and FN is 78. Low values of both FP and FN metrics directly affect the accuracy. Also, the first model from GoogleNet achieved a high sensitivity value because the resulting FN value is small, which directly impacts on sensitivity. The third model of ResNet50 obtained the highest F1-Score value because the resulting FP value does not exceed eight images, impacting the precision. The resulting FP images do not exceed eight images, and subsequently, the F1-Score has a high value.

**Figure 8:**
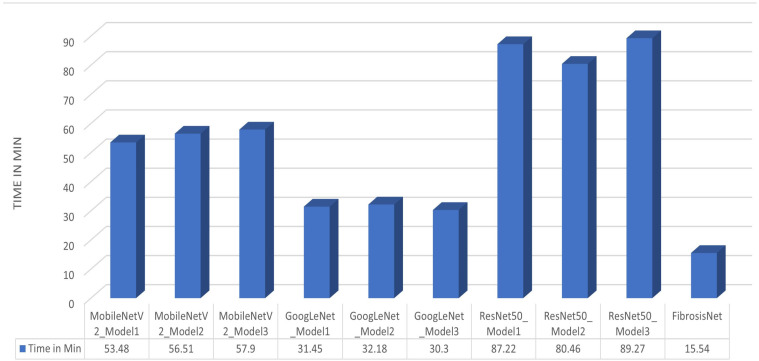
The computation time for each modified model and FibrosisNet.

Regarding the performance time, MobileNet has a minimum value of 53.48 min for model 1. Model 3 has a minimum value of 30.3 min for the GoogleNet, while model 2 has 80.46 min for the ResNet50 network. The FibrosisNet network surpasses all systems since it is consumed in just 15.54 min, indicating less computational complexity, and making our network the best, as described in Figure 8.

Table 8 compares the present study to previous studies in the open literature to demonstrate the pros of the proposed FibrosisNet. It demonstrates that, when compared to previously published research, the proposed approach differs in its high accuracy and capacity for detecting the greatest number of fibrosis images. Furthermore, unlike traditional methods that require manual extraction of features, the given approach can extract features automatically. This can assist clinicians in accurately detecting fibrosis cases and decreasing the chance of errors in detection.

## 5. Conclusions

A novel method for detecting cardiac MRI fibrosis has been provided. The two parts of this method are the identification of cardiac fibrosis and the preprocessing of MRI scans. This depended on the newly developed MRI scans for a better diagnosis. The identification of cardiac fibrosis has been trained using three separate models. The proposed CNN (FibrosisNet) performs the best and shows encouraging results for the new deep learning technique with an accuracy rate of 96.05%, sensitivity of 94.96%, specificity of 97.56%, and F1-Score of 96.54%. For further work, we will use more deep-learning approaches to improve the suggested system’s accuracy in order to assess cardiac fibrosis or myocardial fibrosis by using other modalities to improve the diagnosis system for the patients. For the potential future directions of this study, improving the dimension and the quality of the dataset will lead to better results, developing a complete system that could accurately detect and segment cardiac fibrosis. Overall, this study shows that the healthcare system can be improved for cardiac patients and, hence, several medical complications at early stages can be prevented.

## Figures and Tables

**Table 1:** Summary of some related studies for heart fibrosis, and their accuracy for automated diagnosis.

Reference	DL/ML	Dataset (Patient/ Images)	Target	Algorithm	Performance (%)	Limitations
Campese [26]	ML	642 images	MF detection	CNN Kernel SVM	ACC = 71% SEN = 72%	Lack of accuracy and sensitivity
Dima et al. [27]	ML	Clinical	Detecting myocardial scar	CNN SVM	ACC = 89.22% SEN = 76% SPEC = 87.5%	Unsuitable performance metrics
Zabihollahy and Ukwatta [28]	ML + DL	30 patients	Identification of MF	CNN Cascaded multi-planar U-Net	DSC = 88.61%	Not enough training data
Asif et al. [29]	DL	77 patients	Automated diameter analysis	-	ACC = 71.9%	Less accuracy
Sharkey et al. [30]	DL	1553 patients	Fully automated segmentation	CNN-UNet	ACC = 93%	Lack of training data
Penso et al. [31]	DL	50 patients	Identification of myocardial fibrosis	CNN Tensorflow-Keras	ACC = 71% SEN = 73%	Insufficient sensitivity
Shi et al. [32]	DL	60 patients	Detection ML	ResNext-50	SPEC = 87% SEN = 79% AUC = 83%	Inadequate sensitivity
Jafari et al. [33]	DL	Clinical	LV scar	CTAEM-Net	ACC = 90.18%	Low accuracy
Popescu et al. [34]	DL	2484 images	Scar segmentation	U-Net Res U-Net	ACC 1 = 96% ACC 2 = 75%	Not enough training data
Moccia et al. [35]	DL	250 images	Scar segmentation	FCNNs	SEN = 88.07%	Less Sensitivity
Gumpfer et al. [36]	DL	114 patients	Detecting myocardial scar	CNN + FNN	SPEC = 84.3% ACC = 78.0% SEN = 70.0%	Inappropriate performance metrics
Muthulakshmi and Kavitha [37]	DL	-	Estimate left ventricular volume	CNN + LM	ACC = 86.39%, SEN = 90%	Low accuracy
Ahmed et al. [38]	DL	1041 patients	Automated scar quantification	DCN U-Net	ACC = 82%	lack of testing Low accuracy

**Table 2:** Training and testing data.

**Training Data**		**Testing Data**		**Total Dataset**
Fibrosis	Normal	Fibrosis	Normal	
1536	1200	384	300	3420

**Table 3:** Total learnable parameters.

Layer Order	Layer Name	Output Shape	Learnable Parameters
1	Image Input	[224, 224, 3]	0
2	2D convolution	[222, 222, 10]	760
3	Batch Normalization	[222, 222, 10]	20
4	ReLU	[222, 222, 10]	0
5	2D Max Pooling	[221, 221, 10]	0
6	2D convolution	[219, 219, 10]	2510
7	Batch Normalization	[219, 219, 10]	20
8	ReLU	[219, 219, 10]	0
9	2D Max Pooling	[218, 218, 10]	0
10	2D convolution	[218, 218, 10]	910
11	Batch Normalization	[218, 218, 10]	20
12	ReLU	[218, 218, 10]	0
13	2D Max Pooling	[218, 218, 10]	0
14	Dropout	[218, 218, 10]	0
15	Fully Connected	[1, 1, 2]	950,482
16	SoftMax	[1, 1, 2]	0
17	Classification output	[1, 1, 2]	0
Total Learnable parameters			954,700

**Table 4:** Detailed layer modifications for MobileNetV2.

Model	Layer Order	Old Layer	New Layer	Old Shape	New Shape
Model 1	152	FC (1000 outputs)	FC (2 outputs)	[1, 1, 1000]	[1, 1, 2]
Model 2	151	Average Pooling	ReLU	[1, 1, 1280]	[7, 7, 1280]
	152	FC (1000 outputs)	FC (2 outputs)	[1, 1, 1000]	[1, 1, 2]
Model 3	150	ReLU	FC (20 outputs)	[7, 7, 1280]	[1, 1, 20]
	151	Average Pooling	ReLU	[1, 1, 1280]	[7, 7, 1280]
	152	FC (1000 outputs)	FC (2 outputs)	[1, 1, 1000]	[1, 1, 2]

**Table 5:** GoogleNet layer adjustments for all models.

Model	Layer Order	Old Layer	New Layer	Old Shape	New Shape
Model 1	142	FC (1000 outputs)	FC (2 outputs)	[1, 1, 1000]	[1, 1, 2]
Model 2	141	Dropout	ReLU	[1, 1, 1024]	[1, 1, 1024]
	142	FC (1000 outputs)	FC (2 outputs)	[1, 1, 1000]	[1, 1, 2]
Model 3	140	Average Pooling	FC (35 outputs)	[1, 1, 1024]	[1, 1, 35]
	141	Dropout	ReLU	[1, 1, 1024]	[1, 1, 1024]
	142	FC (1000 outputs)	FC (2 outputs)	[1, 1, 1000]	[1, 1, 2]

**Table 6:** ResNet50 layer modifications for all models.

Model	Layer Order	Old Layer	New Layer	Old Shape	New Shape
Model 1	175	FC (1000 outputs)	FC (2 outputs)	[1, 1, 1000]	[1, 1, 2]
Model 2	174	Average Pooling	ReLU	[1, 1, 2048]	[7, 7, 2048]
	175	FC (1000 outputs)	FC (2 outputs)	[1, 1, 1000]	[1, 1, 2]
Model 3	173	ReLU	FC (20 outputs)	[7, 7, 2048]	[1, 1, 20]
	174	Average Pooling	ReLU	[1, 1, 2048]	[7, 7, 2048]
	175	FC (1000 outputs)	FC (2 outputs)	[1, 1, 1000]	[1, 1, 2]

**Table 7:** Comparison of all provided fined-tuning models with FibrosisNet. The models’ greatest performance has been achieved assigned by bold font.

Network	Modified Model	TP	TN	FP	FN	ACC	SEN (Recall)	SPC	PPV (Precision)	NPV	F1-Score	MCC
**MobileNetV2**	Model 1	359	237	25	63	**87.13%**	**85.07%**	**90.46%**	**93.49%**	**79.00%**	**89.08%**	**73.99%**
	Model 2	363	163	21	137	76.90%	72.60%	88.59%	94.53%	54.33%	82.13%	54.68%
	Model 3	352	209	32	91	82.02%	79.46%	86.72%	91.67%	69.67%	85.13%	63.71%
**GoogleNet**	Model 1	347	259	37	41	**88.60%**	**89.43%**	**87.50%**	**90.36%**	**86.33%**	**89.90%**	**76.82%**
	Model 2	355	237	29	63	86.55%	84.93%	89.10%	92.45%	79.00%	88.53%	72.73%
	Model 3	350	213	34	87	82.31%	80.09%	86.23%	91.15%	71.00%	85.26%	64.20%
**ResNet50**	Model 1	354	243	30	57	87.28%	86.13%	89.01%	92.19%	81.00%	89.06%	74.16%
	Model 2	333	245	51	55	84.50%	85.82%	82.77%	86.72%	81.67%	86.27%	68.49%
	Model 3	376	229	8	71	**88.45%**	**84.12%**	**96.62%**	**97.92%**	**76.33%**	**90.49%**	**77.43%**
**FibrosisNet**		377	280	7	20	**96.05%**	**94.96%**	**97.56%**	**98.18%**	**93.33%**	**96.54%**	**92.02%**

**Table 8:** Comparison of FibrosisNet detection system and other similar works.

Reference	Images	Performance%	Detection Method
Campese [26]	642 images	ACC = 71% SEN = 72%	ML CNN
Dima et al. [27]	–	ACC = 89.22% SEN = 76% SPEC = 87.5%	ML CNN
Popescu et al. [34]	2484 images	ACC 1 = 96% ACC 2 = 75%	U-Net Res U-Net
Moccia et al. [35]	250 images	SEN = 88.07%	FCNNs
Gumpfer et al. [36]	114 patients	84.3%	DL CNN + FNN
**Proposed method** (FibrosisNet)	1140 patients 3420 images	ACC = 96.05% PPV = 98.18%	DL CNN

## Data Availability

The dataset is available upon request.

## References

[1] Amegah A.K. (2018). Tackling the growing burden of cardiovascular diseases in Sub-Saharan Africa. Circulation.

[2] Smith S.C., Collins A., Ferrari R., Holmes D.R., Logstrup S., McGhie D.V., Ralston J., Sacco R.L., Stam H., Taubert K. (2012). Our Time: A call to save preventable death from cardiovascular disease (heart disease and stroke). Circulation.

[3] Chen S.W., Wang S.L., Qi X.Z., Samuri S.M., Yang C. (2022). Review of ECG detection and classification based on deep learning: Coherent taxonomy, motivation, open challenges and recommendations. Biomed. Signal Process. Control.

[4] Alsharqi M., Woodward W.J., Mumith J.A., Markham D.C., Upton R., Leeson P. (2018). Artificial intelligence and echocardiography. Echo Res. Pract..

[5] Cassar A., Holmes D.R., Rihal C.S., Gersh B.J. (2009). Chronic coronary artery disease: Diagnosis and management. Mayo Clin. Proc..

[6] Houssein E.H., Hassaballah M., Ibrahim I.E., AbdElminaam D.S., Wazery Y.M. (2022). An automatic arrhythmia classification model based on improved Marine Predators Algorithm and Convolutions Neural Networks. Expert Syst. Appl..

[7] Narula S., Shameer K., Omar A.M.S., Dudley J.T., Sengupta P.P. (2016). Machine-Learning algorithms to automate morphological and functional assessments in 2D echocardiography. J. Am. Coll. Cardiol..

[8] Kirişli H., Schaap M., Metz C., Dharampal A., Meijboom W., Papadopoulou S., Dedic A., Nieman K., de Graaf M., Meijs M. (2013). Standardized evaluation framework for evaluating coronary artery stenosis detection, stenosis quantification and lumen segmentation algorithms in computed tomography angiography. Med. Image Anal..

[9] Cau R., Solinas C., De Silva P., Lambertini M., Agostinetto E., Scartozzi M., Montisci R., Pontone G., Porcu M., Saba L. (2022). Role of cardiac MRI in the diagnosis of immune checkpoint inhibitor-associated myocarditis. Int. J. Cancer.

[10] Rahman H., Scannell C.M., Demir O.M., Ryan M., McConkey H., Ellis H., Masci P.G., Perera D., Chiribiri A. (2021). High-Resolution cardiac magnetic resonance imaging techniques for the identification of coronary microvascular dysfunction. JACC Cardiovasc. Imaging.

[11] Murtha L.A., Schuliga M.J., Mabotuwana N.S., Hardy S.A., Waters D.W., Burgess J.K., Knight D.A., Boyle A.J. (2017). The processes and mechanisms of cardiac and pulmonary fibrosis. Front. Physiol..

[12] Weber K.T., Sun Y., Bhattacharya S.K., Ahokas R.A., Gerling I.C. (2012). Myofibroblast-mediated mechanisms of pathological remodelling of the heart. Nat. Rev. Cardiol..

[13] Mozaffarian D. (2017). Global scourge of cardiovascular disease. J. Am. Coll. Cardiol..

[14] Moran A.E., Roth G.A., Narula J., Mensah G.A. (2014). 1990–2010 Global Cardiovascular Disease Atlas. Glob. Heart.

[15] St John Sutton M.G., Sharpe N. (2000). Left ventricular remodeling after myocardial infarction. Circulation.

[16] Kim R.J., Fieno D.S., Parrish T.B., Harris K., Chen E.-L., Simonetti O., Bundy J., Finn J.P., Klocke F.J., Judd R.M. (1999). Relationship of MRI delayed contrast enhancement to irreversible injury, infarct age, and contractile function. Circulation.

[17] Ukwatta E., Arevalo H., Rajchl M., White J., Pashakhanloo F., Prakosa A., Herzka D.A., McVeigh E., Lardo A.C., Trayanova N.A. (2015). Image-based reconstruction of three-dimensional myocardial infarct geometry for patient-specific modeling of cardiac electrophysiology. Med. Phys..

[18] Marsan N.A., Bax J.J. (2010). Myocardial fibrosis assessed by CMR to predict events in HCM. Nat. Rev. Cardiol..

[19] Treibel T.A., Fontana M., Steeden J.A., Nasis A., Yeung J., White S.K., Sivarajan S., Punwani S., Pugliese F., Taylor S.A. (2017). Automatic quantification of the myocardial extracellular volume by cardiac computed tomography: Synthetic ECV by CCT. J. Cardiovasc. Comput. Tomogr..

[20] Barragán-Montero A., Javaid U., Valdés G., Nguyen D., Desbordes P., Macq B., Willems S., Vandewinckele L., Holmström M., Löfman F. (2021). Artificial intelligence and machine learning for medical imaging: A technology review. Phys. Medica.

[21] Letourneau-Guillon L., Camirand D., Guilbert F., Forghani R. (2020). Artificial intelligence applications for workflow, process optimization and predictive analytics. Neuroimaging Clin. N. Am..

[22] Avanzo M., Porzio M., Lorenzon L., Milan L., Sghedoni R., Russo G., Massafra R., Fanizzi A., Barucci A., Ardu V. (2021). Artificial intelligence applications in medical imaging: A review of the medical physics research in Italy. Phys. Medica.

[23] Ali M., Ali R. (2021). Multi-Input Dual-Stream Capsule Network for Improved Lung and Colon Cancer Classification. Diagnostics.

[24] Shen D., Wu G., Suk H.-I. (2017). Deep learning in medical image analysis. Annu. Rev. Biomed. Eng..

[25] Haskins G., Kruger U., Yan P. (2020). Deep learning in medical image registration: A survey. Mach. Vis. Appl..

[26] Campese S., Agostini F., Sciarretta T., Pizzi M., Cipriani A., Zanetti M. (2022). Myocardial fibrosis detection using kernel methods: Preliminary results from a cardiac magnetic resonance study. Eur. J. Echocardiogr..

[27] Dima S.-M., Panagiotou C., Mazomenos E.B., Rosengarten J.A., Maharatna K., Gialelis J.V., Curzen N., Morgan J. (2013). On the Detection of Myocardial Scar Based on ECG/VCG Analysis. IEEE Trans. Biomed. Eng..

[28] Zabihollahy F., Rajan S., Ukwatta E. (2020). Machine Learning-Based Segmentation of Left Ventricular Myocardial Fibrosis from Magnetic Resonance Imaging. Curr. Cardiol. Rep..

[29] Asif A., Charters P.F.P., Thompson C.A.S., Komber H.M.E.I., Hudson B.J., Rodrigues J.C.L. (2022). Artificial intelligence can detect left ventricular dilatation on contrast-enhanced thoracic computer tomography relative to cardiac magnetic resonance imaging. Br. J. Radiol..

[30] Sharkey M.J., Taylor J.C., Alabed S., Dwivedi K., Karunasaagarar K., Johns C.S., Rajaram S., Garg P., Alkhanfar D., Metherall P. (2022). Fully automatic cardiac four-chamber and great vessel segmentation on CT pulmonary angiography using deep learning. Front. Cardiovasc. Med..

[31] Penso M., Babbaro M., Moccia S., Baggiano A., Carerj M.L., Guglielmo M., Fusini L., Mushtaq S., Andreini D., Pepi M. (2023). A deep-learning approach for myocardial fibrosis detection in early contrast-enhanced cardiac CT images. Front. Cardiovasc. Med..

[32] Shi K., Li Y., Zhang T.J., Li Z.L., Li H.X., Peng W.L., Xia C.C. (2021). Early Assessment of Myocardial Fibrosis of Hypertrophic Cardiomyopathy with Native-T1-Mapping-Based Deep Learning: A Preliminary Study. J. Sichuan Univ. Med. Sci. Ed..

[33] Jafari M., Shoeibi A., Khodatars M., Ghassemi N., Moridian P., Alizadehsani R., Khosravi A., Ling S.H., Delfan N., Zhang Y.-D. (2023). Automated diagnosis of cardiovascular diseases from cardiac magnetic resonance imaging using deep learning models: A review. Comput. Biol. Med..

[34] Popescu D.M., Abramson H.G., Yu R., Lai C., Shade J.K., Wu K.C., Maggioni M., Trayanova N.A. (2022). Anatomically informed deep learning on contrast-enhanced cardiac magnetic resonance imaging for scar segmentation and clinical feature extraction. Cardiovasc. Digit. Health J..

[35] Moccia S., Banali R., Martini C., Muscogiuri G., Pontone G., Pepi M., Caiani E.G. (2018). Development and testing of a deep learning-based strategy for scar segmentation on CMR-LGE images. Magn. Reson. Mater. Phys. Biol. Med..

[36] Gumpfer N., Grün D., Hannig J., Keller T., Guckert M. (2020). Detecting myocardial scar using electrocardiogram data and deep neural networks. Biol. Chem..

[37] Muthulakshmi M., Kavitha G. Deep CNN with LM learning based myocardial ischemia detection in cardiac magnetic resonance images. Proceedings of the 2019 41st Annual International Conference of the IEEE Engineering in Medicine and Biology Society (EMBC).

[38] Fahmy A.S., Rausch J., Neisius U., Chan R.H., Maron M.S., Appelbaum E., Menze B., Nezafat R. (2018). Automated Cardiac MR Scar Quantification in Hypertrophic Cardiomyopathy Using Deep Convolutional Neural Networks. JACC Cardiovasc. Imaging.

[39] Sandler M., Howard A., Zhu M., Zhmoginov A., Chen L. MobileNetV2: Inverted Residuals and Linear Bottlenecks. Proceedings of the 2018 IEEE Conference on Computer Vision and Pattern Recognition (CVPR), Salt Lake City.

[40] Yilmaz E., Trocan M. (2021). A modified version of GoogleNet for melanoma diagnosis. J. Inf. Telecommun..

[41] Sabour S., Frosst N., Hinton G.E. (2017). Dynamic routing between capsules. arXiv.

[42] He K., Zhang X., Ren S., Sun J. Deep Residual Learning for Image Recognition. Proceedings of the 2016 IEEE Conference on Computer Vision and Pattern Recognition (CVPR).

